# Ultrafast Method for Selective Design of Graphene Quantum Dots with Highly Efficient Blue Emission

**DOI:** 10.1038/srep38423

**Published:** 2016-12-08

**Authors:** Suk Hyun Kang, Sungwook Mhin, Hyuksu Han, Kang Min Kim, Jacob L. Jones, Jeong Ho Ryu, Ju Seop Kang, Shin Hee Kim, Kwang Bo Shim

**Affiliations:** 1 Department of Materials Science and Engineering, Hanyang University, 222 Wangsimri-ro, Seongdonggu, Seoul, 04763, Korea; 2 Korea Institute of Industrial Technology, 113-58, Seohaean-ro, Siheung-si, Gyeonggi-do, 15014, Republic of Korea; 3 Korea Institute of Industrial Technology, Gwahakdanji-ro 137-41, Gangwond-do, 25440, Republic of Korea; 4 Department of Materials Science and Engineering, North Carolina State University, Raleigh, NC 27695, USA; 5 Department of Materials Science and Engineering, Korea National University of Transportation, Chungbuk, 27469, Republic of Korea; 6 Department of Pharmacology & Clinical Pharmacology Lab, College of Medicine, Hanyang University, 222 Wangsimri-ro, Seongdonggu, Seoul, 04763, Korea

## Abstract

Graphene quantum dots (GQDs) have attractive properties and potential applications. However, their various applications are limited by a current synthetic method which requires long processing time. Here, we report a facile and remarkably rapid method for production of GQDs exhibiting excellent optoelectronic properties. We employed the pulsed laser ablation (PLA) technique to exfoliate GQDs from multi-wall carbon nanotube (MWCNTs), which can be referred to as a pulsed laser exfoliation (PLE) process. Strikingly, it takes only 6 min to transform all MWCNTs precursors to GQDs by using PLE process. Furthermore, we could selectively produce either GQDs or graphene oxide quantum dots (GOQDs) by simply changing the organic solvents utilized in the PLE processing. The synthesized GQDs show distinct blue photoluminescence (PL) with excellent quantum yield (QY) up to 12% as well as sufficient brightness and resolution to be suitable for optoelectronic applications. We believe that the PLE process proposed in this work will further open up new routes for the preparation of different optoelectronic nanomaterials.

Great attention has been given to graphene due to its extraordinary physicochemical properties arisen from the unique crystal structure^1^,^2^,^3^,^4^,^5^,^6^, consisting of a monolayered structure of a two-dimensional crystal of sp^2^ carbon atoms arranged in a honeycomb lattice^7^. Graphene shows excellent electronic^8^, thermal^9^, and mechanical properties^10^ with high chemical stability^11^, and graphene-based materials are therefore promising building blocks for future nanotechnologies and nanodevices. In addition, the high intrinsic mobility of graphene^12^ opens the door for potential electronic applications such as photovoltaic cells, supercapacitors, and touch screens^13^,^14^,^15^,^16^,^17^. However, optoelectronic applications utilizing graphene have been limited due to the insufficient on-off current ratio caused by lack of bandgap in pristine graphene^18^. Efforts to engineer the bandgap of graphene led to the development of graphene quantum dots (GQDs)^19^,^20^,^21^,^22^,^23^,^24^,^25^,^26^,^27^,^28^,^29^. In GQDs, the zero-bandgap of graphene can be tuned by the size, shape, and the fraction of sp^2^ domains in sp^3^ matrix, drawing out their quantum confinement^30^,^31^, edge effects^32^,^33^, and thus photoluminescence (PL). Furthermore, the colloidal form of GQDs is more desirable for applications in bioimaging due to their higher followability^33^,^34^,^35^,^36^,^37^,^38^. One of the more exciting potential applications is the use of colloidal GQDs for monitoring activity of cancer cell *in vivo*^39^.

Colloidal GQDs have been prepared mainly via wet-chemical cutting routes using carbon precursors^21^,^22^,^23^. However, most colloidal GQDs synthesized by cutting methods revealed poor optical properties due to a weak visible absorption. GQDs prepared by cutting routes show not only low quantum yields (QY) but also low brightness and resolution of emitting light. Moreover, wet-chemical cuttings are usually performed under the condition with strong acid and base, which requires a costly neutralization process for a long period of time. Several attempts to produce colloidal GQDs thorough a bottom-up strategy have been reported^40^,^41^. A typical bottom-up process utilizes benzene derivatives as carbon precursors in organic solutions where oxidative condensations occur for multiple steps. However, the large scale production of colloidal GQDs is hindered by this time consuming, expensive, and environmentally hazardous process. It is thus necessary to develop a simple, fast, and facile method to produce colloidal GQDs.

Pulsed laser ablation (PLA) is a unique technique to produce nanomaterials on a mass scale^42^. PLA generates highly non-equilibrium conditions in liquids with high temperature and pressure where anomalous reactions and growth of the fragmented species can occur^42^. Recent efforts on laser ablation of highly oriented pyrolytic graphite (HOPG) in water opened the possibility for the preparation of colloidal GQDs^43^ and partially reduced GOQDs (rGOQDs)^44^. However, extremely low QY of GQDs produced via PLA was a problem^43^,^45^, limiting their use for the application such as bioimaging and optoelectronics. Also, an undesired formation of the GOQDs occurred during laser ablation, which causes inconsistent PL properties and thus low resolution of the emitting light due to inhomogeneous size and shape^43^,^45^. Another attempt to produce pristine GQDs from GOQDs thorough harsh thermal reduction process was reported^46^. However, certain amount of oxygenous sites still remained up to a level of 10% in the rGOQDs. These oxygenous functional groups induce defect states within the bandgap, which hinders the clear and distinct blue luminescence of pristine GQDs.

Herein, we propose fast, simple, mild, and cost-effective methodology for production of colloidal pristine GQDs with homogeneous size and shape. In this study, we employed the PLA technique to exfoliate GQDs from multi-walled carbon nanotubes (MWCNTs) as carbon precursors, which will be referred to as the pulsed laser exfoliation (PLE) process. According to our results, the proposed methodology enables selectively producing GQDs and GOQDs by simply changing organic solvents for MWCNTs. More striking is the ability of our method to transform colloidal GQDs from MWCNTs precursors in only 6 min. Size and shape of the GQDs produced by PLE are homogeneous with negligible oxygeneous functional groups. Furthermore, the GQDs emit the distinct blue light with excellent QYs up to 12% at high brightness and resolution, properties that are suitable for biological and optoelectronic applications.

## Results

**Figure 1:**
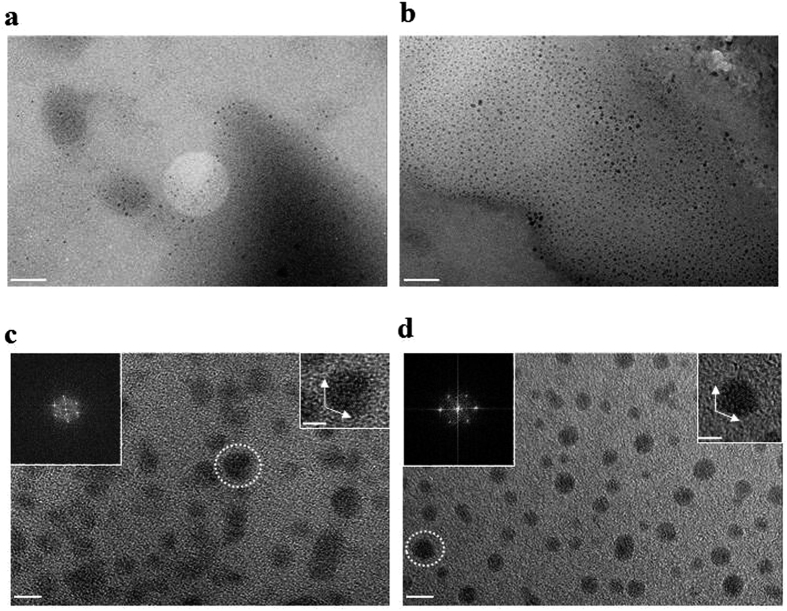
HR-TEM images of e-GOQDs and h-GQDs. (**a**) TEM image of e-GOQDs and (**b**) h-GQDs. They both showing the uniform round shape and size distribution of 1~5 nm. Scale bar 50 nm. (**c**) HR TEM image of e-GOQDs and (**d**) h-GQDs. Insets are the 2D FFT patterns (left). They both show high quality crystalline hexagonal patterns of these quantum dots. Scale Bar 5 nm. Right side insets show the edge structure of e-GOQDs and h-GQDs. Scale bar 2 nm.

Figure 1a,b shows high resolution transmission electron microscopy (HR-TEM) images of GQDs prepared by using laser ablation for 6 min in different mediums. The exfoliated GQDs in both ethanol (e-GOQDs, Fig. 1a) and hexane (h-GQDs, Fig. 1b) have average diameters below 1–5 nm with homogeneous size and shape (supplementary Fig. S1). The height of e-GOQDs and h-GQDs was estimated as 1.2~1.8 nm by using atomic force microscopy (AFM), which corresponds to 3~4 graphene layers (supplementary Fig. S2). Also, according to the FFT patterns (the left insets of Fig. 1c,d), it is confirmed that both types of GQDs are crystallized in the pristine graphene structure. The right insets of Fig. 1c,d show edge structures of both e-GOQDs and h-GQDs, which seem to be parallel to the zigzag orientation^33^. The formation of GQDs with specific edge structure is strongly dependent on how the sp3 carbon domain structure is transformed into the subnanometer sp2 carbon structure. Thus, it is necessary to investigate the exfoliation mechanism of MWCNT to GQD conversion during the PLE process.

**Figure 2:**
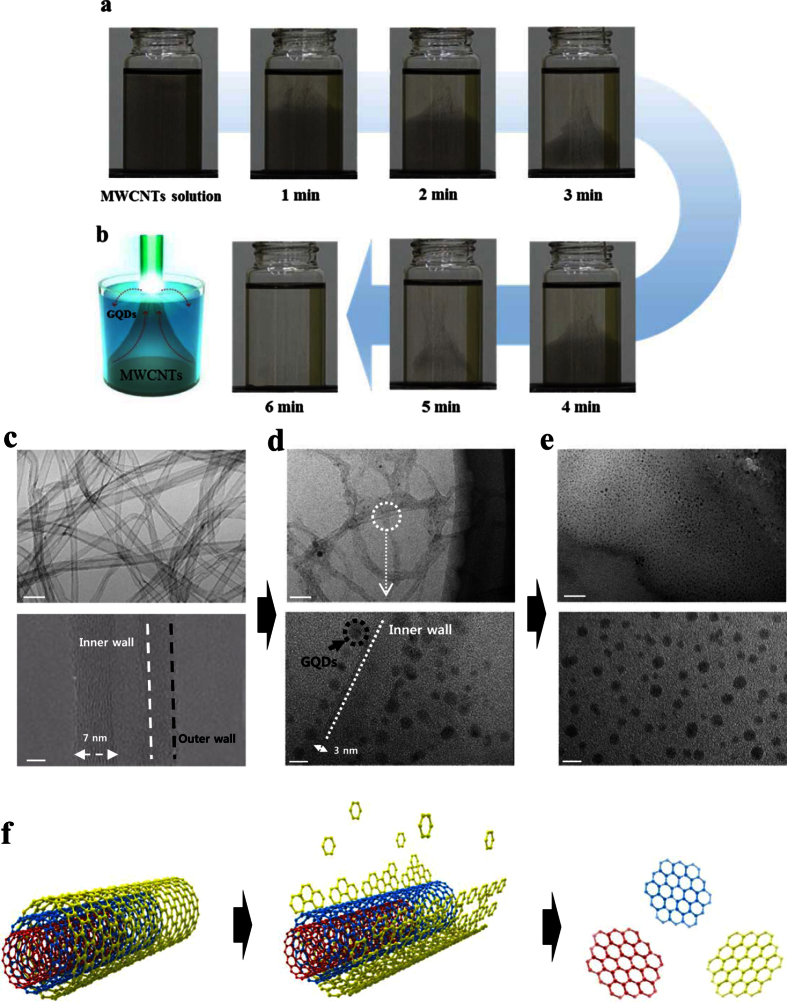
PLE process for ultrafast design of GQDs with highly efficient blue emission. (**a**) Time laps images of the MWCNTs suspension during PLE process. (**b**) Schematic illustration of MWCNTs suspension during PLE process showing cone-shape convection. HR TEM images of MWCNTs **(c**) before laser ablation, (**d**) after laser irradiated 1 min, (**e**) 6 min, respectively. Scale bar 50 nm (top TEM images), Scale bar 5 nm (bottom HR-TEM images) (**f**) Schematic illustration of possible mechanism for the exfoliation MWCNTs to GQDs.

GQDs were completely exfoliated from MWCNTs using by PLE within 6 minutes (See supplementary Movie S1 in real time). The MWCNTs suspension was rapidly changed from black to transparent after laser irradiation. The suspension clearly turns transparent within 6 min, which demonstrates that MWCNTs transformed to GQDs in extremely shorter time scales than other processes. Within the first 1 min of irradiation, the original black suspension turns transparent from top to bottom (Fig. 2a). As the irradiation time increases up to 2 min, almost half of the suspension becomes transparent. The transparent suspension after laser irradiation indicates that the exfoliated GQDs can form colloids with the liquid medium. At this stage, MWCNTs are dispersed in the medium with a cone-shape (Fig. 2b). The black color was faded away near at the neck of cone compared to the body (Fig. 2a). We speculated that the faded black color near at the neck might be attributed to the partially exfoliated MWCNTs during PLE (supplementary Fig. S3). In order to verify this, we carefully investigated the microstructure evolution of MWCNTs at different PLE steps via HR-TEM. HR-TEM images of the MWCNTs at each step (PLE process for 1 min, 3 min, and 6 min) are shown in Fig. 2c,d,e, respectively. MWCNTs before laser irradiation have tubular structure with diameter of 15~20 nm (Fig. 2c). Characteristic inner and outer walls of the MWCNT structures are clearly observed in the HR-TEM images at this initial stage. After laser irradiation, the tubular structure of MWCNTs were partially destructed and small black dots were observed along line with the edges of the tubes (Fig. 2d). The HR-TEM image confirms that the small black dots are the exfoliated GQDs from the outer wall of MWCNTs. At this stage, the wall thickness of MWCNTs was reduced from 7~8 nm to about 3 nm. These GQDs are arrayed parallel to the inner walls which still maintain the tubular structure. Upon further irradiation up to 6 min, the inner walls of the MWCNTs also broke into GQDs, apparent because the tubular structure of MWCNTs completely disappear and only GQDs with homogeneous shape and size remain (Fig. 2e). Figure 2f depicts the proposed mechanism for exfoliation and transformation of MWCNTs to GQDs during the PLE process. The outer walls of MWCNTs are firstly exfoliated to GQDs while inner walls of MWCNTs remain unbroken. These exfoliated GQDs (formed from outer walls of MWCNTs) likely form along the edges of inner tubes. The partially broken MWCNTs might move vertically in the suspension while producing cone-shape convection (Fig. 2b). With further irradiation, the tubular structure of MWCNTs is completely transformed to GQDs colloid with the liquid medium. The yield of PLE process was calculated by dividing the weight of dried e-GOQDs and h-GQDs product by the weight of the starting MWCNTs. Based on this method, the yield of e-GOQDs and h-GQDs were determined as 14 and 12 wt%, respectively.

**Figure 3:**
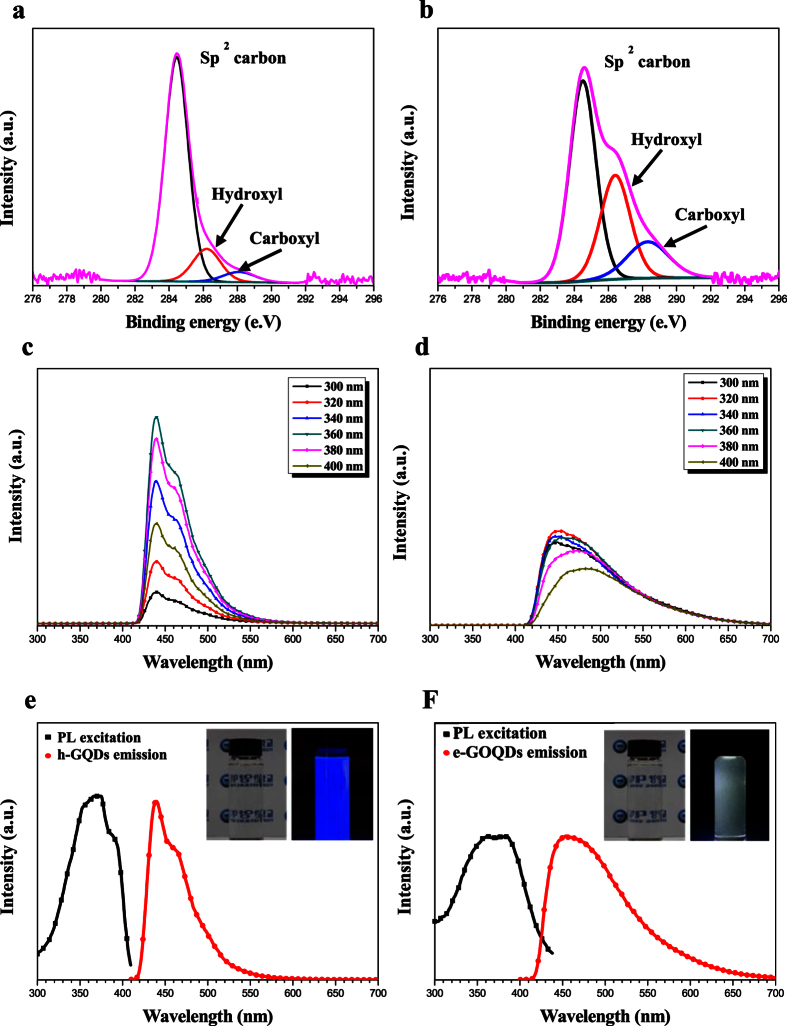
XPS spectra and optical properties of h-GQDs and e-GOQDs. XPS spectra for (**a**) h-GQDs and (**b**) e-GOQDs. PL spectra excited at different wavelengths for (**c**) h-GQDs and (**d**) e-GOQDs. PL emission spectra of (**e**) h-GQDs and (**f**) e-GOQDs. Insets of (**e**) and (**f**) are the digital images of h-GQDs and e-GOQDs under excitation 360 nm, respectively.

X-ray photoelectron spectroscopy (XPS) was carried out in order to investigate the chemical bonding of the h-GQDs and e-GOQDs, respectively. XPS spectra of both h-GQDs and e-GOQDs are illustrated in Fig. 3a,b. XPS spectra of the h-GQDs exhibit a dominant peak at 284.5 eV which can be assigned to the representative sp2 C1s peak^22^,^33^ (Fig. 3a). Negligible sp3 carbon peaks from oxygeneous functional groups such as hydroxyl (287.0 eV) or carboxyl (289.5 eV) were detected^27^ (Fig. 3b). According to quantitative analysis of the XPS spectra, fractions of sp2 and sp3 carbon peaks are determined as 81.3% and 18.7%, respectively (supplementary Table S1). In contrast to h-GQDs, e-GOQDs show a significantly increased fraction of sp3 carbon peaks from oxygeneous functional groups. Quantitative fractions are calculated as 53.19% and 46.81% for sp2 and sp3 carbon peaks, respectively. This implies that, strictly speaking, the GQDs that are exfoliated from MWCNTs dispersed in ethanol are not GQDs but GOQDs (referred to as e-GOQDs)^27^. Thus, GQDs and GOQDs can be selectively produced via PLE process simply changing the medium for MWCNTs carbon precursor.

We investigated the optical properties of h-GQDs and e-GOQDs exfoliated from MWCNT, respectively. Figure 3c,d shows photoluminescence (PL) spectra of h-GQDs under the range of the excitation wavelength between 300 nm and 400 nm. In Fig. 3c, the distinct blue emission of h-GQDs is clearly represented irrespective to the excitation wavelength. As the excitation wavelength changed from 300 nm to 360 nm, the peak intensity was gradually increased. However, peak intensity of the emission was abruptly decreased when excitation wavelength exceeded 360 nm. Contrary to this, the PL spectra of e-GOQDs exhibited a broad emission peak with a long tail existing up to 700 nm (Fig. 3d), implying the clear blue emission of pristine graphene might not occur from e-GOQDs. Also, the emission peak position and intensity of the e-GOQDs were not significantly varied at different excitation wavelengths, indicating that the PL spectra of the e-GOQDs are independent of the wavelength for excitation. The PL emission spectra of both the h-GQDs and e-GOQDs were recorded under the excitation wavelength of 360 nm. It can be seen in Fig. 3e that the PL excitation spectra of h-GQDs have the strongest excitation peak at 360 nm with the shoulder near at 380 nm, while e-GOQDs exhibited the broader excitation peak at 360 nm (Fig. 3f). Broader PL excitation spectra of e-GOQDs might be related with the oxygeneous functional groups emitting PL spectra between 300 and 440 nm. The insets of Fig. 3e,f show the h-GQD and e-GOQD nanocolloids under the excitation wavelength of 360 nm. The brightness and resolution of the blue emission from h-GQDs is better than that of e-GOQDs, i.e. higher brightness and improved resolution. Also, the PLE spectrum of e-GOQDs shows intensity at 360 nm, while the UV-visible absorption of e-GOQDs shows a broad absorption spectrum (supplementary Fig. S4b)^27^. Contrary to e-GOQDs, as shown in Supplementary Fig. S4a, the characteristic absorbance peak (350 nm) was clearly observed for h-GQDs near the wavelength of PL emission peak (350 nm), which implies existence of the energy level within the bandgap corresponding to the blue emission of GQDs^38^. The QY was calculated for both h-GQDs and e-GOQDs. Details for QY calculations are explained in Electronic Supporting Information (ESI). The result indicates that the QY of h-GQDs is about 12%, which is much larger than those of e-GOQDs (0.8%) and the excellent optical properties than other wet-chemical routes (Supplementary Table S2). The high QY as well as the improved resolution and brightness of the emitting blue PL are distinguishable optical properties of the PLE-synthesized h-GQDs, demonstrating that the PLE process is an effective way to prepare high quality GQDs for bioimaging and optoelectronic applications.

## Discussion

A possible mechanism for the exfoliation of MWCNTs to h-GQDs or e-GOQDS via laser exfoliation is proposed in Fig. 2f. In order to exfoliate h-GQDs or e-GOQDs from MWCNTs via laser exfoliation, C-C chemical bonds should be broken by interaction of the laser with the MWCNTs. During PLE process, the laser can interact with matter via two different phenomena, a photolytic and/or a pyrolytic process^47^. The former process (photolytic) breaks chemical bonds of the material with the aid of photon energy and can directly convert matter without thermal interaction. The latter (pyrolytic) induces thermal heating, melting, or evaporation of the material when the laser interacts with matter. The photolytic process dominantly occurs at the target surface. However, the pyrolytic process can be more pronounced in the bulk of the target^47^ (supplementary Fig. S5). The absorbed energy transfers into bulk of the target via thermal conduction resulting in heating, melting, or evaporation of the exfoliated material. Thus, the exfoliated material from the target obtained via a pyrolytic process might have more inhomogeneous shape and size distributions as compared to those obtained via a photolysis process. Inhomogeneous shape and size distributions of the exfoliated material obtained via the pyrolytic process might be due to partial destruction and grain growth of the exfoliated material possibly arisen from the thermal effects.

The use of MWCNTs with ultrathin carbon walls provides a benefit of minimizing the pyrolytic process during laser exfoliation. The irradiated laser can interact with ultrathin carbon walls of MWCNTs via mainly photolytic process. Also, the tubular structure of MWCNTs can effectively suppress thermal conduction of the absorbed energy into the bulk. For the purpose of comparison, we also performed the PLE process using a bulky carbon precursor. Supplementary Fig. S6 shows HR-TEM images for the exfoliated GQDs using graphite in ethanol. It is clearly seen in Supplementary Fig. S6, that the exfoliated GQDs from graphite have spherical shape with particle size (10~50 nm). The size distribution for GQDs exfoliated from MWCNTs and graphite are shown in supplementary Figures S1b,d and S6d, respectively. Much broader size distribution of GQDs exfoliated from graphite implies that pyrolytic as well as photolytic processes can occur when bulky carbon material is used in the PLE process. The stability of the e-GOQDs and h-GQDs colloids was tested by measuring PL emission for the as-prepared samples and samples aged for 1–2 months (supplementary Figure S7). The results show that no change in the PL intensities of the e-GOQDs and h-GQDs is observed even after aging for 2 months, implying the e-GOQDs and h-GQDs prepared in this work remain stable for at least 2 months. Thus, it is concluded that for achieving ultrafast fabrication of GQDs with homogenous size distribution, MWCNTs should be preferred as the carbon precursor over bulky carbon materials such as graphite and graphene oxide. The tubular structure of MWCNTs, which is composed of ultrathin carbon outer walls with a hollow inner part, can effectively minimize the pyrolytic process during PLE process.

In conclusion, we successfully prepared GQDs and GOQDs with homogeneous shape and size in an extremely short time (less than 6 min) using MWCNTs as the carbon precursor in a PLE process. GQDs and GOQDs can be selectively produced by simply changing liquid mediums (i.e., hexane and ethanol). The exfoliated GQDs from MWCNTs exhibited superior optical properties such as high QY and improved brightness and resolution compared to the counterparts of GOQDs. The exfoliation mechanism of GQDs from MWCNTs was investigated using HR-TEM. It was found that GQDs are initially exfoliated from the outer walls of the MWCNTs. Subsequently, the partially broken MWCNTs can be dispersed in the medium, forming a cone-shape convection during further transformation to GQDs. During the PLE process, interaction of the laser with MWCNTs occurs via photolytic process rather than pyrolytic process thanks to the thin walled characteristic of the MWCNTs. The thin walls of the MWCNTs enable minimizing the thermal interaction between laser and precursor, which can lead to homogeneous size distribution of the exfoliated GQDs. We expect that the proposed method in this work will significantly reduce cost and time for the formation of the high quality GQDs with excellent optical properties. In addition, the PLE process can further broaden the range of application for the preparation of different optoelectronic nanomaterials.

## Methods

### Sample Preparation

MWCNTs were prepared by catalyst chemical vapor deposition (CCVD) method which has purity up to 99.9%^48^. The MWCNT has a tubular structure with a diameter of 15~20 nm and a length of 0.5~10 μm. 0.005 g of as-prepared MWCNTs was dispersed in 500 ml of n-hexane (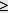
99%, Sigma Alrich) and ethanol (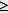
99.5%, Sigma Alrich) mediums, respectively. Ultrasonication was subsequently performed on the solutions for 2 h in order to achieve homogeneous dispersion of MWCNTs. 50 ml of the solution was transferred into 50 ml glass vials, and then the PLE process was performed on the fixed vials for 6 min using a Quanta Ray – LAB 190 equipment (spectra physics, USA). The laser was focused on the center of vials with a focal length of 10 cm and a spot diameter of 10 mm^2^, respectively. For specifying laser characteristics, the wavelength was 355 nm, the power density was 3.82 W/cm^2^, the energy was 100 mJ/pulse, the repetition rate was 30 Hz, the pulse width was 10 nm, and the maximum output was 2.5 J, respectively.

### Sample Characterizations

HR-TEM images were taken using a 2100 F field emission gun TEM (JEM 2100 F, JEOL, USA, 200 KV) for GQDs and GOQDs samples. XPS spectra were recorded for the both samples using VG ESCALAB 220i (Thermo scientific, USA). XPS survey and high resolution scans were performed with the pass energies of 100 eV and 20 eV, respectively. X-ray beam size was approximately 100 μm. GQDs and GOQDs samples for XPS measurement were prepared via spin coating technique. Silicon (Si) substrate was used for spin coating. Rotation speed was adjusted to 3,000 rpm. The samples were dried at room temperature for 2 hrs before the measurement. Room temperature PL spectra of GQDs and GOQDs were collected using a photoluminescence spectrophotometer (PerkinElmer, LS55 with 100 mW laser diode, USA) in the wavelength range from 400 nm to 700 nm. The excitation wavelength used for measuring PL emission spectra was 430 nm.

## Additional Information

**How to cite this article**: Kang, S. H. *et al*. Ultrafast Method for Selective Design of Graphene Quantum Dots with Highly Efficient Blue Emission. *Sci. Rep.* **6**, 38423; doi: 10.1038/srep38423 (2016).

**Publisher's note:** Springer Nature remains neutral with regard to jurisdictional claims in published maps and institutional affiliations.

## Supplementary Material

Supplementary Information

Supplementary Video
