# Supplementary material for: Ultrafast Method for Selective Design of Graphene Quantum Dots with Highly Efficient Blue Emission
Source: Sci Rep. 2016 Dec 8;6:38423. doi: 10.1038/srep38423 (PMC5144005; doi:10.1038/srep38423)
Supplement: Supplementary Information [file srep38423-s1.pdf]

Supplementary information

Ultrafast Method for Selective Design of Graphene Quantum Dots with Highly Efficient Blue Emission

Suk Hyun Kang[1*], Sungwook Mhin[2*], Hyuksu Han[3*], Kang Min Kim[4], Jacob L. Jones[5], Jeong Ho Ryu[6] , Ju Seop Kang[7], Shin Hee Kim[7] & Kwang Bo Shim[1].

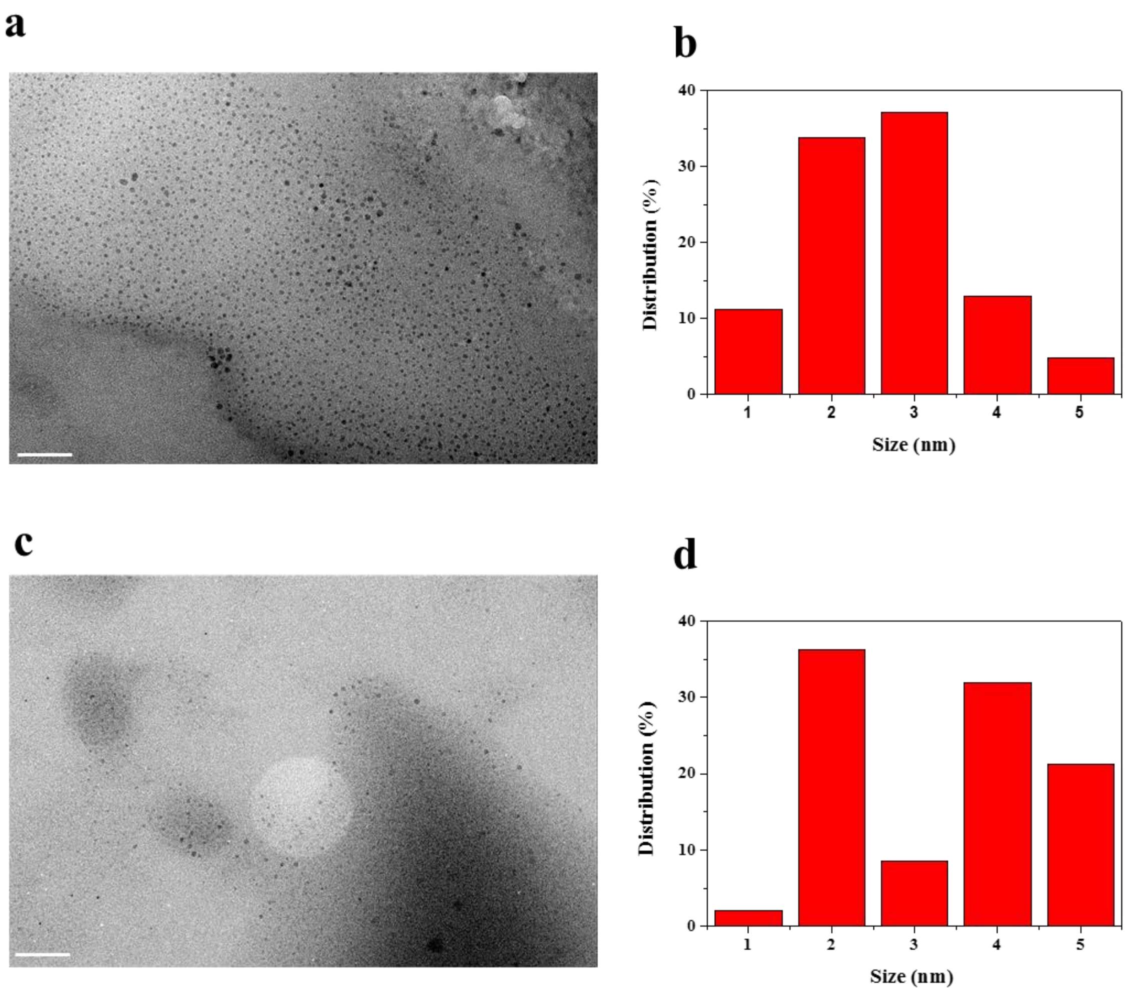

Supplementary Figure S1. TEM characterization of e-GOQDs and h-GQDs. a, The TEM images of h-GQDs. Scale bar, 50 nm **b,** The size distribution histogram of h-GQDs. **c,** The TEM images of e-GOQDs. Scale bar, 50 nm **d,** The size distribution histogram of e-GOQDs

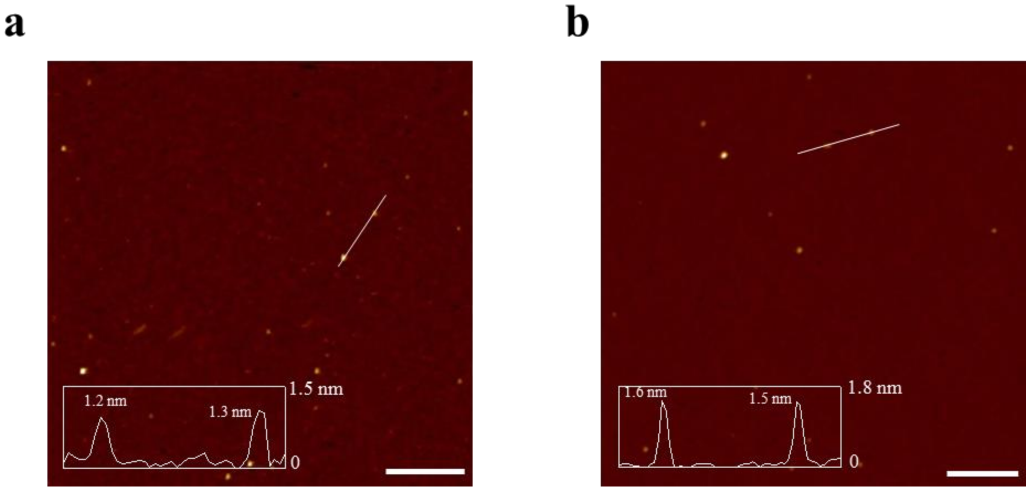

Supplementary Figure S2 . AFM images of a, e-GOQDs and b, h-GQDs. Scale bar, 1 μm

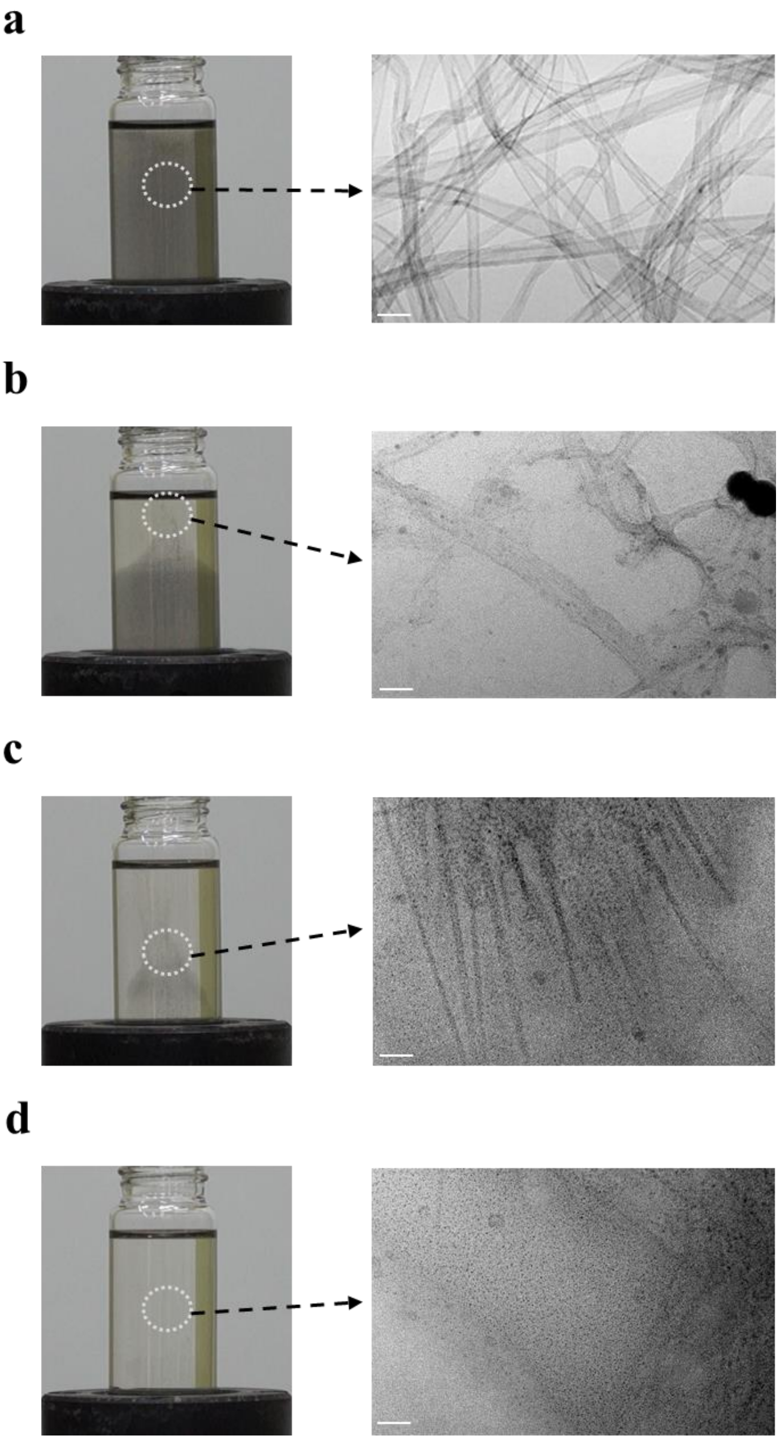

Supplementary Figure S3. HR TEM images of partially exfoliated MWCNTs during laser ablation. a, before laser exfoliation **b,** 2 min **c,** 5min **d,** after laser exfoliation. Scale bar, 50 nm

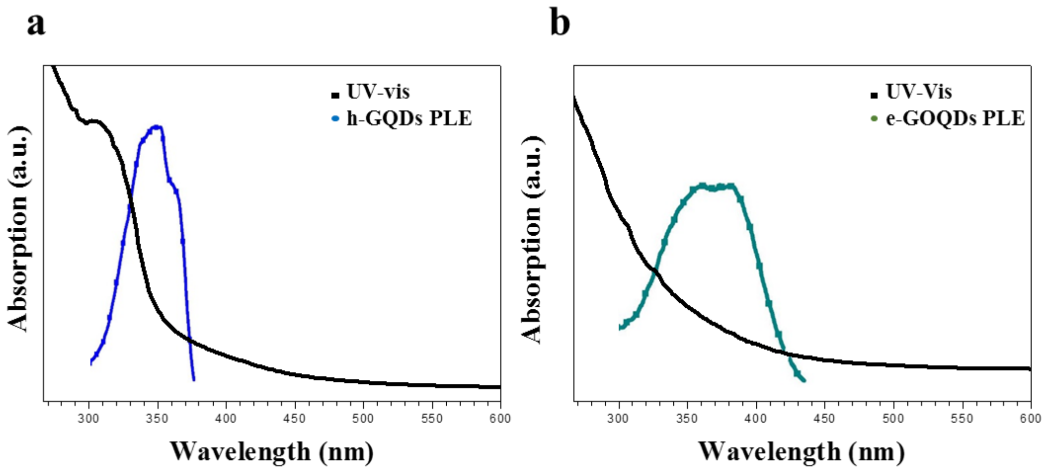

Supplementary Figure S4. Comparison between UV-visble absorbance and PL-excitation (PLE). a, h-GQDs. **b,** e-GOQDs

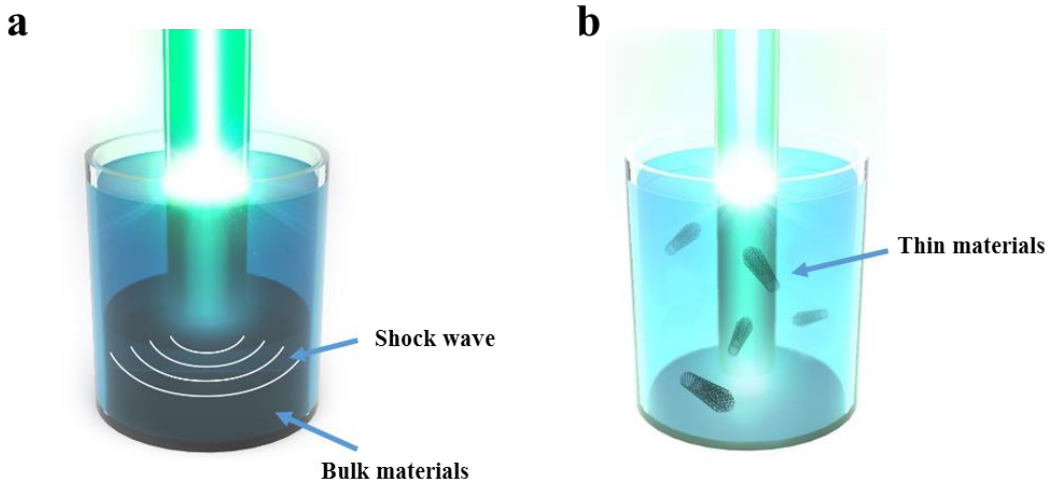

Supplementary Figure S5. Schematic illustration of the during laser process. a, pyrolytic process. **b,** photolytic process

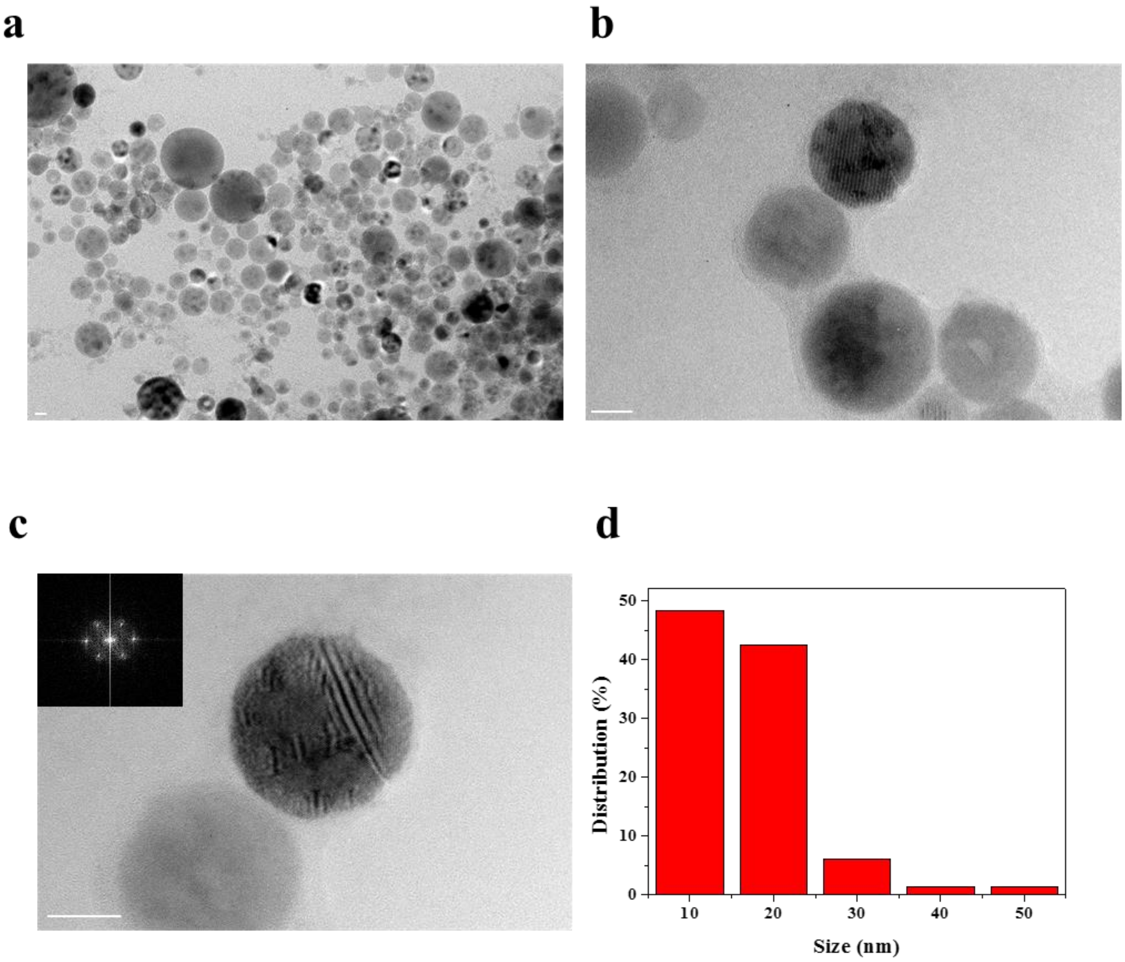

Supplementary Figure S6. TEM characterization of g-GQDs using graphite as carbon precursors in ethanol. a-c, The TEM images scale bar 10 nm. **d,** The size distribution histogram.

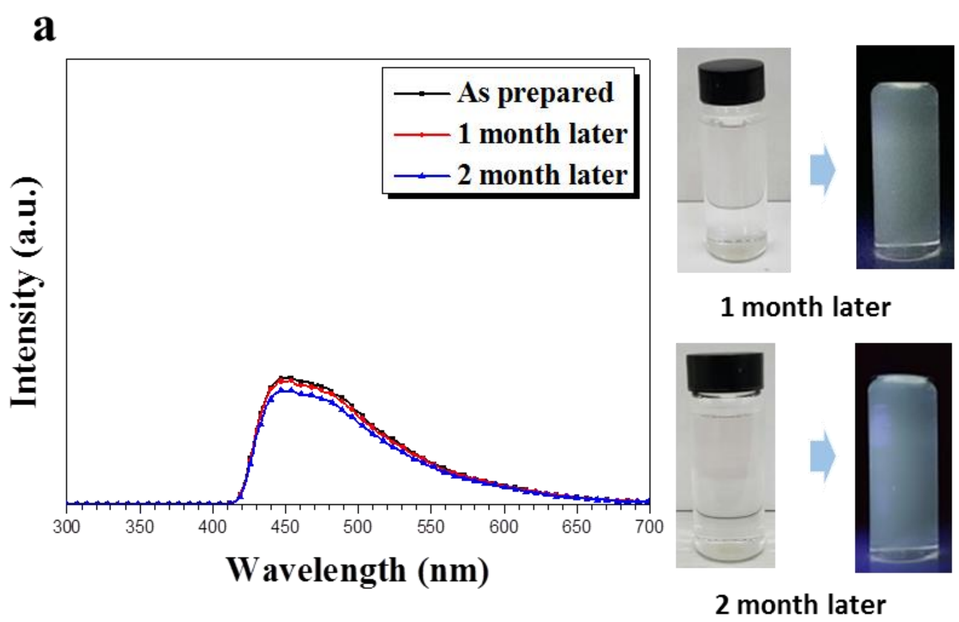

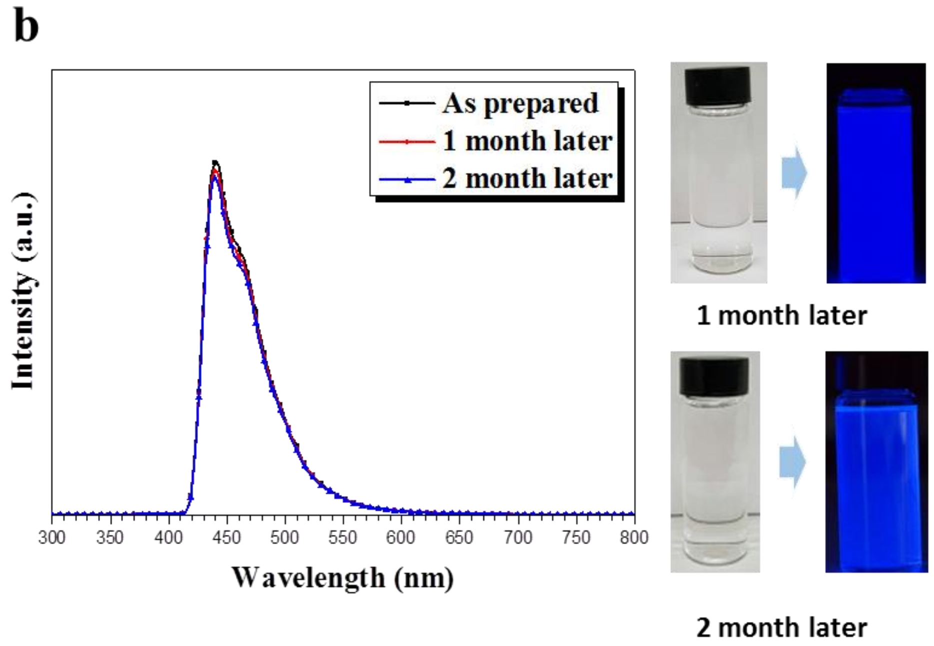

Supplementary Figure S7. Time dependent PL emission spectra and digital images (under 360 nm excitation). a, e-GOQDs, **b,** h-GOQDs

	Peak Binding Energy	h-GQDs	e-GOQDs
C=C and C-C (%)	284.6	81.3	53.19
Hydroxyl, Carboxylate carbon group	286-288.5	18.7	46.81

Supplementary Table S1. XPS analysis of h-GQDs, e-GOQDs

Methods	Subclassfication	Starting materials	Processing time	Color	Ref.
Top-down	PLE	MWCNTs	~ 6 min	Blue	In this work
	Acidic oxidation	GO	A few days	Blue	51
		GO	A few days	Blue	49
		Carbon black	24 hr	Green	17
	Hydrothermal	GO	A few days	Blue	10
		GO	~ 26 hr	Green	62
		RGO	~ 18 hr	Blue	69
	Solvothermal	GO	~ 6 hr	Green	16
		GO	~ 8 hr	Blue to Green	85
	Microwave	GO	~ 5 hr	Green, Blue	6
	Microwave – Hydrothermal	GO	~ 5 min	Blue	65
	Ultrasonic chemistry	-	~ 12 hr	Blue	12
	Electrochemistry	-	~ several min	Green	53
Bottom – up	Precusor pyrolsysis	Glucose	~ 11 min	DUV, Blue	11
		Citric acid	~ 3 hr	Blue	55
	Pyrolysis and Exfoliation	Unsubstituted HBC	~ 24 hr	Blue	47

Supplementary Table S2. A summary of GQDs synthesized via general synthetic method

Supplementary Movie S1. The process of exfoliated MWCNTs suspension using by PLE.
